# Epidemiological investigation of infectious occupational exposure among dental students in a stomatological teaching hospital: a cross-sectional study

**DOI:** 10.3389/fpubh.2025.1562112

**Published:** 2025-07-24

**Authors:** Na Tang, Si He, Sha Li

**Affiliations:** Changsha Stomatological Hospital, Changsha, China

**Keywords:** stomatology department, dental student, infectious occupational exposure, hospital infection, occupational exposure

## Abstract

**Purpose:**

To analyze the infectious occupational exposure status and related factors in stomatological hospital students.

**Methods:**

The infectious occupational exposure data reported by students in Changsha Stomatological Hospital from 2019 to 2023 were collected and analyzed from three aspects: basic situation, exposure situation and emergency treatment after exposure.

**Results:**

A total of 1,151 students were investigated from 2019 to 2023, and 48 of them had infectious occupational exposure, with an occupational exposure rate of 4.17%. Interns (6.44%) had the highest rate of occupational exposure to infectious diseases, and most of the exposures were related to handling medical waste (62.50%) and puncture wounds by hollow needles (70.83%). Maxillofacial surgery (54.17%) was the department with the highest proportion of students with infectious occupational exposure. The investigation showed that although none of the exposed students were infected after follow-up, 56.25% of the students still did not receive proper emergency treatment after infectious occupational exposure, and 16.67% of the students did not receive timely report after exposure.

**Conclusion:**

Dental students have a high risk of occupational exposure to infectious diseases, so it is very important to strengthen their occupational protection awareness and operational skills, standardize the emergency treatment measures and reporting procedures of students after occupational exposure, and ensure the correct treatment and reporting of dental students after occupational exposure.

## Introduction

As an old Chinese saying goes, “Toothache may not be a disease, but the pain can feel worse than one.” Oral health has become an increasingly concerned health issue. Although dental procedures are performed in relatively small areas of the mouth, the high-speed rotation of dental instruments during treatment can generate large amounts of aerosols, which may contain bacteria, fungi, and viruses (1), and potentially dispersing throughout the entire room (2). Microorganisms in aerosols can also proliferate on dental equipment and water pipes, forming biofilms on surfaces. If sterilization is not thorough, the bacteria and other microorganisms in the biofilm may continue to generate infectious aerosols during subsequent treatments (3), potentially exposing dental healthcare workers to a prolonged infectious environment. Furthermore, dental instruments are predominantly delicate and sharp handheld tools, thereby increasing the occupational exposure risk for dental healthcare workers (4). Research has shown that dental students are highly susceptible to needle stick injuries and may also encounter numerous incidents of exposure to patients’ bodily fluids (5). The quality of infection control education in dental teaching institutions is crucial and indispensable for reducing student exposure. However, it has been observed that the majority of studies focusing on student occupational exposure have relied on self-designed questionnaires for data collection (6), which are likely to be affected by recall bias and may not fully capture the circumstances surrounding students’ occupational exposures (7). Based on the reported cases of occupational exposure among dental students, this study retrospectively examines the five-year occupational exposure rate among students at a tertiary-grade A stomatological hospital. It analyzes the current status and causes of occupational exposure among dental students, identifies key risk factors, and proposes targeted preventive measures. The findings aim to provide reference for dental hospitals and teaching institutions in safeguarding students’ occupational health.

## Materials and methods

### Research object

Selecting Changsha Stomatological Hospital in Hunan Province, China as the research site, the hospital is an affiliated dental hospital of Hunan University of Traditional Chinese Medicine and one of the main dental teaching institutions in Hunan Province. It provides clinical training to hundreds of dental students every year. The students who participate in clinical training at this hospital are mainly divided into four categories (Interns: senior students majoring in dentistry who come to the hospital for internships; Postgraduates: graduate students who pursue a master’s degree in clinical dentistry at this hospital. Physicians receiving standardized training (PRST): students who receive standardized training for resident physicians at this hospital. Advanced-Study students: students who receive training outside the national enrollment plan to improve their oral professional level). Four types of dental students from 2019 to 2023 were selected as the research subjects, and occupational exposure related data that occurred and were reported within 5 years were collected. This is a populational epidemiological study, no animal experiments are involved, no human clinical studies are included, and there are no potentially identifiable images or data, so it is not applicable for ethical approval. However, the person in charge of this study has made a record to the Science and Education department of the Changsha Stomatological Hospital, so as to ensure the authenticity and confidentiality of the data in this study. All research subjects were anonymous.

Inclusion criteria: interns, postgraduates, physicians receiving standardized training (PRST) and Advanced-Study students who have completed clinical training in Changsha Stomatological Hospital.

Exclusion criteria: (1) students who are unable to complete the one-year follow-up due to dropout or other reasons; (2) students who are only short-term interns in clinical practice and do not involve clinical operations.

### Research methods

The data were collected by combining the active reporting of infectious occupational exposure and the follow-up of nosocomial infection management department. Dental students are required to complete an occupational exposure registration form immediately after occupational exposure and report it to the infection management department of the hospital, and professional doctors should assess and dispose of their exposure and follow-up (8). All students who registered and were eligible were selected. We selected all participating eligible students and did not miss or intentionally select any student. This study primarily compiles data based on the reported cases of occupational exposure among students and the completed Healthcare Worker Occupational Exposure to Infectious Diseases Case Registration Form. It mainly includes three aspects: basic information (gender, age, occupational category, and department), exposure information (exposure process, exposure site, exposure method, and exposure source), and post-exposure treatment and reporting information (washing time, whether disinfection is required, whether emergency treatment is correct, post-exposure preventive measures, whether infection occurs after exposure and report relevant information). To facilitate subsequent follow-up on whether dental students become infected after occupational exposure, they report incidents using their real names when completing the Infectious Disease Occupational Exposure Registration Form. Therefore, if the reported data is incomplete, S. H. (responsible for data collection and management) will contact the student to request supplementary information. However, during the data aggregation and analysis phase, this staff member will remove all identifying information before compiling the data for the analysis team. Consequently, the data used for statistical analysis in the study are anonymized.

### Statistical methods

The description of count data was expressed by absolute number (N) and relative number (rate or component ratio, %). Chi-square test was used for comparison between groups, and the statistical test level was 0.05. All the analyses employed IBM SPSS Statistics version 20.

## Results

### Basic characteristics

**Figure 1:**
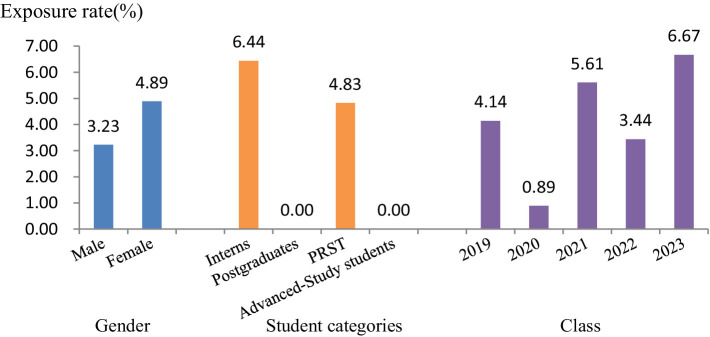
Comparison of occupational exposure rates among stomatology students from 2019 to 2023 (PRST, physicians receiving standardized training).

A total of 1,151 students from four categories were investigated from 2019 to 2023, among which 48 students had infectious occupational exposure, with an occupational exposure rate of 4.17%. The basic characteristics of each year are shown in Table 1. The data indicate an occupational exposure rate of 3.23% among male students and 4.89% among female students. Statistical analysis revealed no significant difference between the two groups (*χ*^2^ = 1.946, *p* = 0.163). As the *p*-value exceeds 0.05, the current findings do not support the conclusion that female students exhibited a higher occupational exposure rate than male students in this study. But there were significant differences in occupational exposure rates among students of different categories (*χ*^2^=24.401, *p* = 0.000) and students of different grades (*χ*^2^ = 11.685, *p* = 0.020) (Figure 1).

**Table 1:** Characteristics of stomatology students with infectious occupational exposure.

Class	Number (exposure rate, %)	Gender (male/female)	Age (years)
2019	7/169(4.14)	3/4	22–23
2020	2/225(0.89)	0/2	21–22
2021	11/196(5.61)	3/8	20–25
2022	10/291(3.44)	3/7	19–26
2023	18/270(6.67)	7/11	20–26
Total	48/1151(4.17)	16/32	19–26

### Characteristics of occupational exposure processes

Among all the links of occupational exposure, the highest proportion of occupational exposure occurred in the disposal of medical waste (62.50%), the most frequently affected site was the index finger (66.67%), the highest proportion of exposure equipment was the hollow needle (70.83%), and the highest proportion of exposure source was the blood (93.75%). Among all clinical departments of stomatology, oral and maxillofacial surgery accounted for the highest proportion of students’ occupational exposure (54.17%), followed by oral emergency department (16.67%). The above differences were statistically significant, and specific characteristics were shown in Table 2.

**Table 2:** Clinical procedures involved with infectious occupational exposures among stomatology students.

Item	Number	Constituent ratio	*χ*^2^	*P*
Procedure			54.889	0.000
During the treatment	12	25.00		
Disposal of wastes	30	62.50		
Local anesthesia	5	10.42		
Other	1	2.08		
Exposure site			63.778	0.000
Thumb	10	20.83		
Index finger	32	66.67		
Middle finger	5	10.42		
Palm	1	2.08		
Exposed instruments			45.750	0.000
Hollow needle puncture	34	70.83		
Solid needle puncture	8	16.67		
Sharp cut	6	12.50		
Exposure source			73.500	0.000
Blood	45	93.75		
Saliva	3	6.25		
Workplace			80.451	0.000
Dept. of Oral and Maxillofacial Surgery	26	54.17		
Dept. of Oral Emergency	8	16.67		
Dept. of Endodontics	7	14.58		
Dept. of Periodontology	1	2.08		
Dept. of Orthodontics	2	4.17		
General department or Outpatient department	2	4.17		
Others	2	4.17		

### Post-occupational exposure treatment and reporting

According to the “Guidelines for the Protection of Occupational exposure to blood-borne Pathogens,” after blood-borne occupational exposure, medical staff should use soap solution and running water to clean the contaminated parts. When the mucosa such as the eye is contaminated, a large amount of normal saline should be used to wash the mucosa repeatedly. When there is a wound, gently squeeze the wound from the proximal end to the distal end to avoid squeezing the wound area, squeeze out the blood at the injured area as much as possible, then rinse the wound with soapy water and running water, disinfect and bandage the wound area with 75% ethanol or 0.5% iodophor. If the exposure source is a patient with blood-borne infectious disease or a suspected patient, corresponding post-exposure preventive and blocking measures should be taken (9).

Among the students with occupational exposure investigated in this study, 41.67% of the students washed the wound for less than 3 min after exposure, 14.58% of the students did not carry out disinfection, and 56.25% of the students failed to carry out correct emergency treatment. After the evaluation by professional doctors, 7 persons (14.58%) received hepatitis B immunization measures, and 1 person (2.08%) received HIV infection blocking measures. Through the follow-up of the hospital infection management department, all occupational exposure workers were not infected after occupational exposure. However, 16.67% of the students failed to report the situation to the hospital infection management department in time after occupational exposure (Table 3).

**Table 3:** Emergency treatment and report analysis of stomatology students after infectious occupational exposure.

Item	Number	Constituent ratio	$\chi^{2}$	*P*
Flushing time (min)			14.250	0.001
<3	20	41.67		
3 ~ 5	6	12.50		
≥5	22	45.83		
Disinfect			48.167	0.000
Yes	41	85.42		
No	7	14.58		
Correct emergency handling			1.500	0.221
Yes	21	43.75		
No	27	56.25		
Preventive measure			82.688	0.000
Hepatitis B immunization	7	14.58		
HIV blocking	1	2.08		
No	40	83.33		
Post-exposure infection			96.000	0.000
Yes	0	0.00		
No	48	100.00		
Reported in time			42.667	0.000
Yes	40	83.33		
No	8	16.67		

## Discussion

This study found that although the total number of female students in the stomatology hospital studied and the number of infectious occupational exposure were more than that of males, the gender exposure rate of males and females was not statistically significant, which was consistent with the results of Mungure EK’s study (10). In terms of occupational categories, interns have the highest occupational exposure rate, which is consistent with the findings of Ravi A (4), and oral students and inexperienced practitioners are the most vulnerable to occupational exposure to needle stick injuries. According to the clinical experience of this study, this may be because interns are often assigned to do the final sorting work of contaminated instruments in clinical practice, and their professional skills are not developed and their clinical experience is limited, so they are easy to be scratched by sharp instruments such as needles, probes or drills during the final treatment of medical waste (11). This is in line with the finding in this study that instrument disposal accounts for the highest proportion of occupational expose-related operations. A 10-year monitoring study also showed that occupational exposure in a dental teaching environment was mostly associated with instrument cleaning (12).

A large number of dental students have experienced one or more occupational exposures during their training, among which percutaneous injuries are the majority, and needle-stick injury is the most common source of exposure (13). Similarly, this study also found that needle-stick injury (hollow needle or solid needle) was the most common way of occupational exposure among students, accounting for 87.5%, and the exposure sites were almost concentrated in the thumb and index finger, which may have been caused by failure to comply with safety techniques such as single-handed needle recapping or failure to use safety-engineered devices (14). Although the dental department may appear to have more contact with the patient’s saliva, more occupational exposure occurs from the patient’s blood, and studies have shown that a minor oral surgery can lead to undetectable blood contamination of clinical surfaces and the physician’s personal protective equipment (15–17). Due to the large number of oral microvessels and the fragile oral environment of most patients, procedures often lead to bleeding. This is especially pronounced in maxillofacial surgery. Maxillofacial surgery operations mostly involve invasive operations such as tooth extraction, and bleeding is difficult to avoid, so medical staff are more likely to be exposed to the blood environment of patients. This study also proves that students in maxillofacial surgery have a higher proportion of occupational exposure than those in other departments.

Proper emergency management and preventive measures are key to avoiding infection in exposed persons. A systematic review also found that most studies showed inadequate reporting of needle-stick injuries and knowledge of post-exposure management in dental students (18). This study found that less than half of the stomatology students with occupational exposure would wash the wound for more than 5 min at the first time, and only 43.75% of the students would use the correct emergency treatment, Consistent with Wu L’s research (19), most students believe that the education they receive about occupational exposure is inadequate. With a sound occupational exposure disposal system and emergency treatment process, the School of Stomatology can strengthen the education of students on the transmission of blood-borne pathogens, so that medical personnel can make post-exposure treatment more clearly after occupational exposure, and pay more attention to the necessity of occupational exposure reporting (20). Raising awareness is significantly associated with reducing the incidence of needle-stick injury, and correct awareness plays a key role in reducing the incidence of needle-stick injury in dental students (21). Timely, correct and effective preventive measures taken after occupational exposure can reduce the injury of occupational exposure to a certain extent and minimize its impact (22). Therefore, the relevant departments of the hospital should improve the emergency treatment and reporting process system of occupational exposure (23), integrate the publicity and implementation of relevant standards into the hospital sense management training plan, and do pre-job training. Clinical departments are regularly organized to carry out situational occupational exposure emergency drills, and through repeated emergency drills, medical personnel are helped to realize the importance of occupational protection and post-exposure emergency treatment from the ideological perspective.

The study also has some limitations. First of all, this study is a cross-sectional investigation, which can only provide some occupational exposure clues, and cannot draw causal links. Secondly, the occupational exposure information case form filled in by occupational exposed persons contains limited contents, and some factors that may lead to students’ occupational exposure, such as daily working hours and number of patients received, are not covered. In the future, more detailed occupational exposure information case forms can be designed and filled in immediately when students have occupational exposure, so as to report exposure information more accurately and truthfully.

In summary, among students majoring in stomatology, young interns have the highest rate of occupational exposure to infectious diseases, and medical waste disposal is an important link in their occupational exposure. The key to preventing occupational exposure of stomatological students is to formulate a complete occupational exposure handling and reporting process, strengthen the training of emergency handling of occupational exposure for stomatological students, especially maxillofacial surgery students, improve their occupational protection ability and correct operational skills, and avoid needle-stick injury.

## Data Availability

The original contributions presented in the study are included in the article/supplementary material, further inquiries can be directed to the corresponding author.

## References

[1] Kumbargere Nagraj S Eachempati P Paisi M Nasser M Sivaramakrishnan G Verbeek JH. Interventions to reduce contaminated aerosols produced during dental procedures for preventing infectious diseases. Cochrane Database Syst Rev. (2020) 10:CD013686. doi: 10.1002/14651858.CD013686.pub2, PMID: 33047816 PMC8164845

[2] Rautemaa R Nordberg A Wuolijoki-Saaristo K Meurman JH. Bacterial aerosols in dental practice – a potential hospital infection problem? J Hosp Infect. (2006) 64:76–81. doi: 10.1016/j.jhin.2006.04.011, PMID: 16820249 PMC7114873

[3] Barbeau J. Waterborne biofilms and dentistry: the changing face of infection control. J Can Dent Assoc. (2000) 66:539–41. PMID: 12584771

[4] Ravi A Shetty PK Singh P Wakode D Modica SF Kodaganallur Pitchumani P. Needlestick injuries in dentistry: time to revisit. J Am Dent Assoc. (2023) 154:783–94. doi: 10.1016/j.adaj.2023.06.004, PMID: 37530693

[5] Huang J Gan Y Xu H Li N An N Cai Z. Prevalence and characteristics of needlestick injuries among dental interns during their first-year clinical training: an observational study. BMC Oral Health. (2023) 23:194. doi: 10.1186/s12903-023-02892-5, PMID: 37009865 PMC10067515

[6] Wicker S Rabenau HF. Occupational exposures to bloodborne viruses among German dental professionals and students in a clinical setting. Int Arch Occup Environ Health. (2010) 83:77–83. doi: 10.1007/s00420-009-0452-3, PMID: 19626335

[7] Huang J Li N Xu H Jiang Y Guo C Li T. Epidemiology of needlestick injury exposures among dental students during clinical training in a major teaching institution of China: a cross-sectional study. J Dent Sci. (2022) 17:507–13. doi: 10.1016/j.jds.2021.07.018, PMID: 35028077 PMC8740099

[8] National Health Commission of the People’s Republic of China. Standard for healthcare associated infection surveillance WS/T312—2023. Chin J Infect Control. (2023) 9:1129–42.

[9] National Health Commission of the People’s Republic of China. Guideline for prevention and control for occupational exposure to bloodborne pathogen GBZ/T 213–2008. Beijing: People's Medical Publishing House (2009).

[10] Mungure EK Gakonyo JM Mamdani Z Butt F. Awareness and experience of needle stick injuries among dental students at the University of Nairobi. Dental Hospital East Afr Med J. (2010) 87:211–4. doi: 10.4314/eamj.v87i5.63076, PMID: 23057284

[11] Zachar JJ Reher P. Percutaneous exposure injuries amongst dental staff and students at a university dental clinic in Australia: a 6-year retrospective study. Eur J Dent Educ. (2022) 26:288–95. doi: 10.1111/eje.12701, PMID: 34117686

[12] Younai FS Murphy DC Kotelchuck D. Occupational exposures to blood in a dental teaching environment: results of a ten-year surveillance study. J Dent Educ. (2001) 65:436–48. PMID: 11425248

[13] Stewardson DA Palenik CJ McHugh ES Burke FJ. Occupational exposures occurring in students in a UK dental school. Eur J Dent Educ. (2002) 6:104–13. doi: 10.1034/j.1600-0579.2002.00253.x, PMID: 12269865

[14] Tabassum N Rida Dimashkieh M Chowdary Jasthi V Murdhi AlEnazi F Mohamed Mostafa Kamal A Kumar SM. A simple technical innovation to prevent needle stick injuries among dental professionals. Eur Rev Med Pharmacol Sci. (2024) 28:1733–40. doi: 10.26355/eurrev_202403_35586, PMID: 38497855

[15] Al-Eid RA Ramalingam S Sundar C Aldawsari M Nooh N. Detection of visually imperceptible blood contamination in the Oral surgical clinic using forensic Luminol blood detection agent. J Int Soc Prev Community Dent. (2018) 8:327–32. doi: 10.4103/jispcd.JISPCD_10_18, PMID: 30123765 PMC6071351

[16] Kannan KV Jr Kandhasamy S John RR Chinnakutti S. Detection of visually imperceptible blood contamination in the surgical area using Luminol among different Oral surgical procedures: An observational study. Cureus. (2024) 16:e53821. doi: 10.7759/cureus.53821, PMID: 38465148 PMC10924243

[17] Bergmann N Lindörfer I Ommerborn MA. Blood and saliva contamination on protective eyewear during dental treatment. Clin Oral Investig. (2022) 26:4147–59. doi: 10.1007/s00784-022-04385-1, PMID: 35165772 PMC8853203

[18] Huang J Li N Xu H Liu Y An N Cai Z. Global prevalence, risk factors, and reporting practice of needlestick and sharps injuries among dental students: a systematic review and meta-analysis. J Hosp Infect. (2022) 129:89–101. doi: 10.1016/j.jhin.2022.06.015, PMID: 35781020

[19] Wu L Yin YL Song JL Chen Y Wu YF Zhao L. Knowledge, attitudes and practices surrounding occupational blood-borne pathogen exposure amongst students in two Chinese dental schools. Eur J Dent Educ. (2016) 20:206–12. doi: 10.1111/eje.12162, PMID: 26184829

[20] Myers JE Myers R Wheat ME Yin MT. Dental students and bloodborne pathogens: occupational exposures, knowledge, and attitudes. J Dent Educ. (2012) 76:479–86. PMID: 22473560

[21] Thekkiniyakath Ali AS Alsour N Almansour AS Albahlal A Alahmari H Alrumi F. The knowledge, attitude, and perception of Needlestick injuries among dental students in Riyadh, Kingdom of Saudi Arabia: a cross-sectional survey. Cureus. (2023) 15:e50939. doi: 10.7759/cureus.50939, PMID: 38249216 PMC10800081

[22] Robinson P. Sharps injuries in dental practice. Prim Dent Care. (1998) 5:33–9. PMID: 9526266

[23] Saleem H Waly N Abdelgawad F. Knowledge, attitude, and practice (KAP) of post exposure prophylaxis for fifth year dental students at a private Egyptian university: a cross-sectional study. BMC Oral Health. (2023) 23:167. doi: 10.1186/s12903-023-02890-7, PMID: 36964540 PMC10039496

